# Supplementary material for: The G3BP1-UPF1-Associated Long Non-Coding RNA CALA Regulates RNA Turnover in the Cytoplasm
Source: Noncoding RNA. 2022 Jun 30;8(4):49. doi: 10.3390/ncrna8040049 (PMC9326601; doi:10.3390/ncrna8040049)
Supplement: Supplementary file 1 [file ncrna-08-00049-s001.zip › ncrna-1754440-supp fig.pdf]

non-coding RNA

MDPI

Supplemental figures

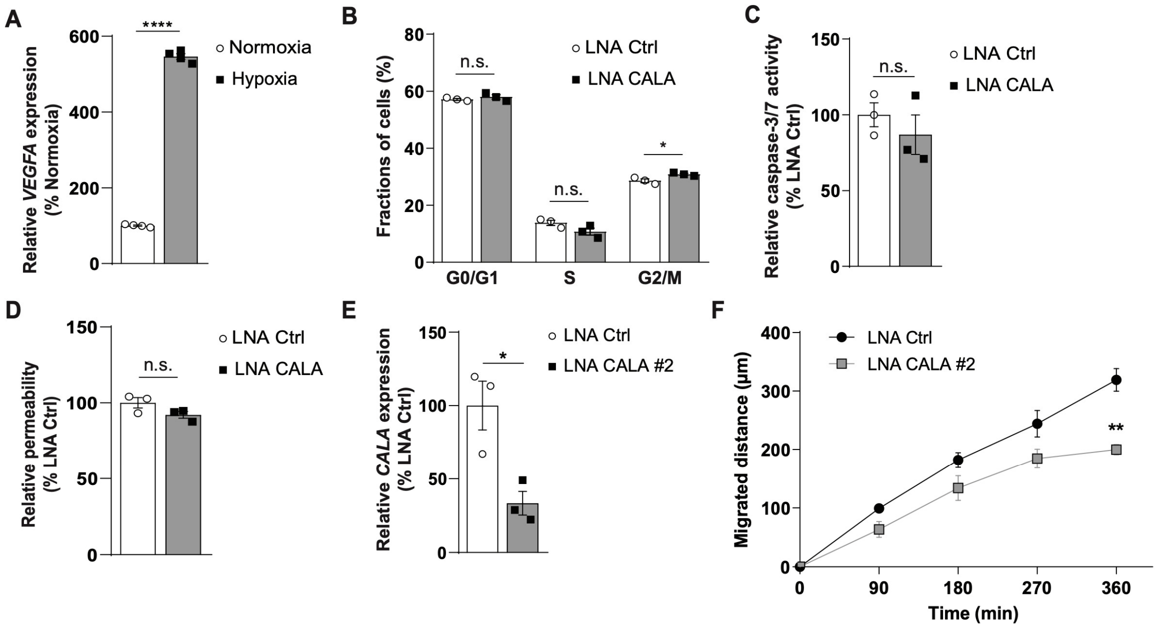

Figure S1. Stimuli-responsive lncRNA *CALA* regulates endothelial sprouting and migration. (A) Relative *VEGFA* expression in HUVECs upon hypoxia (24h, 0.2% O_2, n=4) determined by RT-qPCR. Analysis of (B) cell cycle progression, (C) caspase-3/7 activity, and (D) endothelial permeability in HUVECs upon *CALA*-silencing compared to controls (n=3). (E) *CALA* expression after LNA-mediated silencing in HUVECs determined by RT-qPCR (n=3). (F) Migratory capacity of HUVECs upon *CALA* silencing compared to controls (n=3). Data information: In A-F data are represented as means ± SEM and derive from independent biological replicates, *p < 0.05, **p < 0.01, ****p < 0.0001, n.s.: non-significant. A-F: two-tailed unpaired t-test.

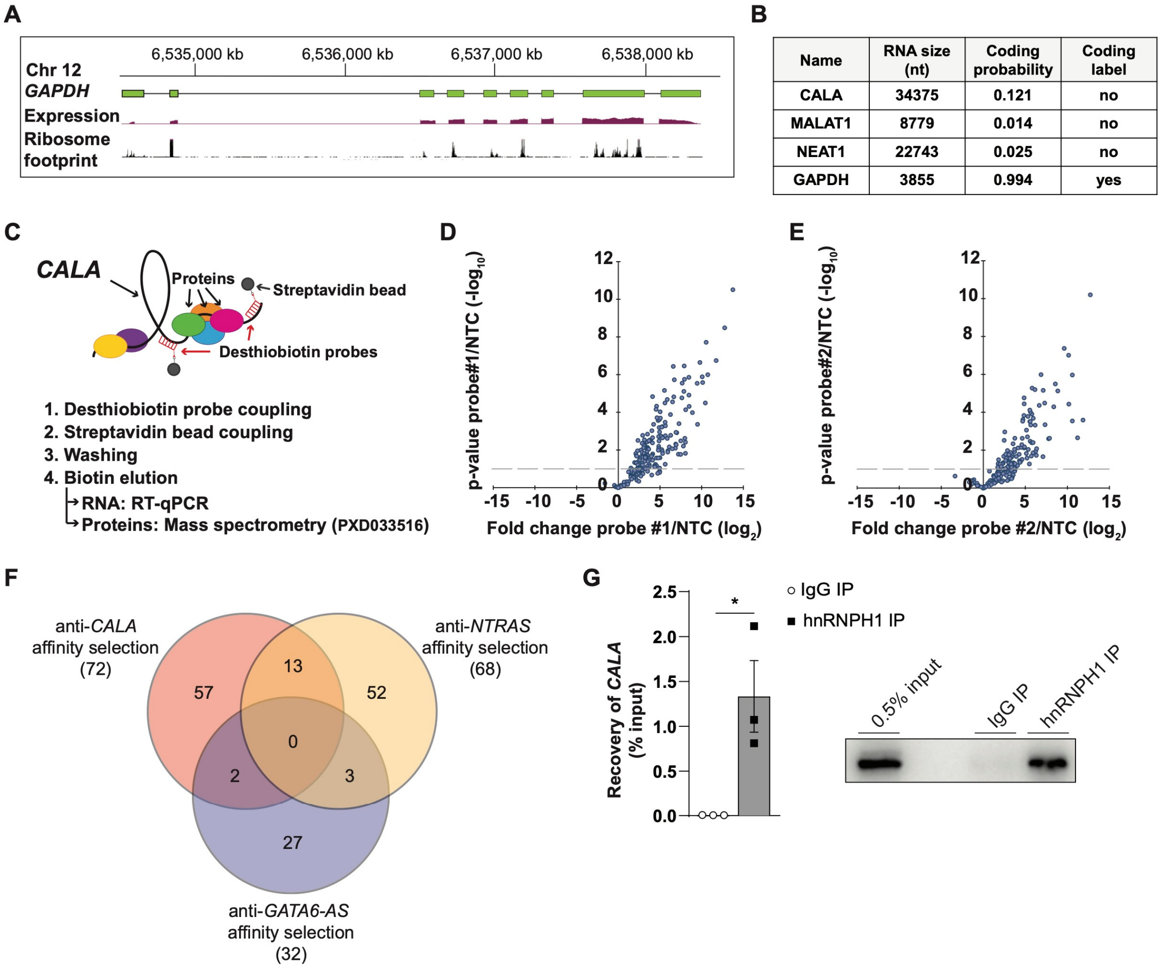

Figure S2. ***CALA*** **interacts with multiple RNPs and primarily associates with cytoplasmic G3BP1.** (A) *GAPDH* gene locus showing ribosome footprinting (GWIPs-viz-riboseq tracks) and aortic expression (GTEx V8 RNA-seq). (B) Coding-potential analysis of indicated transcripts using the Coding-Potential Assessment Tool. (C) Schematic representation of antisense affinity selection of endogenous *CALA*-RNPs. (D-E) Volcano plot of proteins identified *via* mass spectrometry in *CALA* antisense-affinity selection using *CALA*-targeting probe (D) #1 or (E) #2. Dashed line indicating threshold of $p < 0.05$. (F) Venn diagram showing the overlap of significantly enriched proteins from *CALA*, *NTRAS*, and *GATA6-AS* affinity selections. (G) Relative enrichment of *CALA* in anti-hnRNPH1 RIPs determined by RT-qPCR (n=3). Representative western blot shown. Data information: In G data are represented as means ± SEM and derive from independent biological replicates, *$p < 0.05$. D,E,G: two-tailed unpaired t-test.

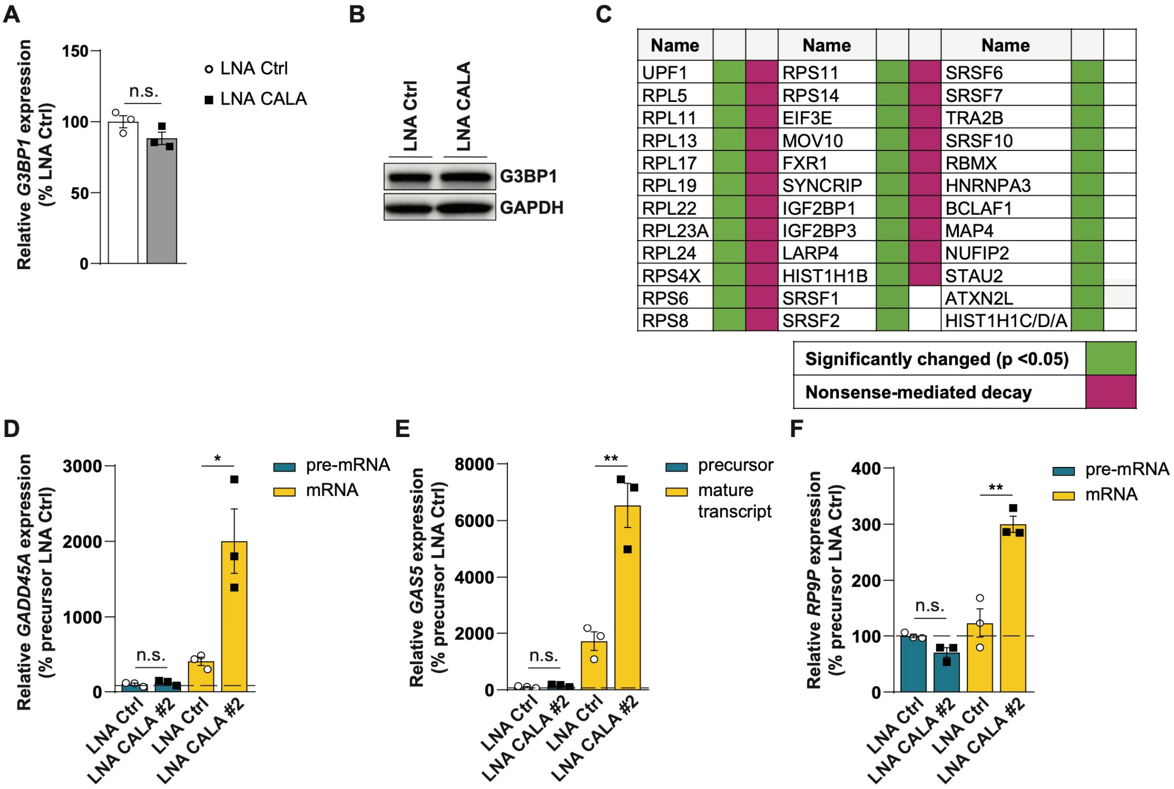

Figure S3. *CALA* impacts the composition and integrity of cytoplasmic G3BP1-RNPs driving mRNA decay. (A) Relative mRNA expression of G3BP1 in control and *CALA*-silenced HUVECs determined by RT-qPCR (n=4). (B) Representative western blot of G3BP1 protein expression upon *CALA* silencing. (C) Identity of significantly changed proteins in G3BP1-co-immunoprecipitations upon *CALA* silencing.(D-F) Relative expression of (D) *GADD45A*, (E) *GAS5*, and (F) *RP9P* pre-mRNA and mRNA upon silencing of CALA determined by RT-qPCR (n=3). Data information: In A, D-F data are represented as means ± SEM and derive from independent biological replicates, *p < 0.05, **p < 0.01, n.s.: non-significant. A, D-F: two-tailed unpaired t-test.
